# Vitamin D deficiency as a predictor of poor prognosis in patients with acute respiratory failure due to COVID-19

**DOI:** 10.1007/s40618-020-01370-x

**Published:** 2020-08-09

**Authors:** G. E. Carpagnano, V. Di Lecce, V. N. Quaranta, A. Zito, E. Buonamico, E. Capozza, A. Palumbo, G. Di Gioia, V. N. Valerio, O. Resta

**Affiliations:** 1 Institute of Respiratory Disease, Department of Basic Medical Science, Neuroscience, and Sense Organs, University of Bari “Aldo Moro”, piazza Giulio Cesare 11, 70125 Bari, Italy; 2 Pneumology Department, “Di Venere” Hospital Bari, Bari, Italy; 3 Cardiology Department, “SS Annunziata” Hospital, Taranto, Italy

**Keywords:** COVID-19, Vitamin D deficiency, Acute respiratory failure, Mortality risk

## Abstract

**Purpose:**

Hypovitaminosis D is a highly spread condition correlated with increased risk of respiratory tract infections. Nowadays, the world is in the grip of the Coronavirus disease 19 (COVID 19) pandemic. In these patients, cytokine storm is associated with disease severity. In consideration of the role of vitamin D in the immune system, aim of this study was to analyse vitamin D levels in patients with acute respiratory failure due to COVID-19 and to assess any correlations with disease severity and prognosis.

**Methods:**

In this retrospective, observational study, we analysed demographic, clinical and laboratory data of 42 patients with acute respiratory failure due to COVID-19, treated in Respiratory Intermediate Care Unit (RICU) of the Policlinic of Bari from March, 11 to April 30, 2020.

**Results:**

Eighty one percent of patients had hypovitaminosis D. Based on vitamin D levels, the population was stratified into four groups: no hypovitaminosis D, insufficiency, moderate deficiency, and severe deficiency. No differences regarding demographic and clinical characteristics were found. A survival analysis highlighted that, after 10 days of hospitalization, severe vitamin D deficiency patients had a 50% mortality probability, while those with vitamin D ≥ 10 ng/mL had a 5% mortality risk (*p* = 0.019).

**Conclusions:**

High prevalence of hypovitaminosis D was found in COVID-19 patients with acute respiratory failure, treated in a RICU. Patients with severe vitamin D deficiency had a significantly higher mortality risk. Severe vitamin D deficiency may be a marker of poor prognosis in these patients, suggesting that adjunctive treatment might improve disease outcomes.

## Introduction

Vitamin D deficiency and insufficiency is a worldwide condition, involving both adults and children, whose association with metabolic, autoimmune and infectious comorbidities has been extensively studied [1]. In particular, several studies highlighted a link between vitamin D deficiency and an increased risk of respiratory tract infections. Chalmers et al. demonstrated that patients with bronchiectasis and vitamin D deficiency were more likely to be chronically colonised with bacteria and had higher airway inflammation than patients with sufficient levels of vitamin D [2]. In a recent study conducted by Mamani et al., a low serum level of 25 hydroxyvitamin D (25(OH)D) was associated with a higher incidence of community-acquired pneumonia and more severe disease [3]. Moreover, vitamin D deficiency is common in critically ill patients and associates with adverse outcomes, as found by Dancer et al. in a cohort of patients with acute respiratory distress syndrome (ARDS). In that study, higher levels of vitamin D were detected in survivors of ARDS then in non-survivors, suggesting that supplementation of vitamin D might also have value as treatment for ARDS [4].

Vitamin D is a fat-soluble vitamin produced from 7-dehydrocholesterol due to the action of UVB radiation and subsequently converted to 25(OH)D in the liver and then to the active form (calcitriol 1, 25(OH)D) in the kidneys or other organs. In addition to being involved in bone metabolism, facilitating the absorption of calcium and phosphorus from the intestinal tract, the role of vitamin D in the immune system has been studied in vitro [5–7]. A recent review analyzed the mechanisms by which vitamin D reduces the risk of microbial infections. It stimulates innate cellular immunity, through the induction of antimicrobial peptides, such as cathelicidins, IL-37 and defensins. It also inhibits the cytokine storm, reducing the production of pro-inflammatory cytokines such as IFNγ and TNFα. Finally, it modulates the adaptive immune response, suppressing the Th1 response and promoting cytokines production by Th2 cells [8].

Nowadays, the world is experiencing a pandemic caused by infection with the severe acute respiratory syndrome coronavirus 2 (SARS-CoV-2) [9]. Coronavirus disease 19 (COVID-19) is a respiratory tract infection whose clinical manifestations vary from mild to severe disease, sometimes requiring admission to intensive care (ICU) due to development of ARDS or sepsis [10, 11]. The cause of this extreme variability in clinical manifestations has been researched in disease pathogenesis. In a recent study conducted in a Wuhan Hospital in January 2020, it has been noted that COVID-19 patients had high concentrations of IL1B, IFNγ, IP10, and MCP1, probably leading to activated T-helper-1 (Th1) cell responses, and that these levels were higher in patients requiring ICU admission. These data suggest that the cytokine storm was associated with disease severity [12]. Therefore, the possibility of using anti-inflammatory and immunomodulating therapies in COVID 19 patients is gaining increasing attention.

In our experience conducted in a Respiratory Intermediate Care Unit (RICU), we found a high prevalence of vitamin D deficiency and insufficiency in a cohort of patients with moderate-to-severe acute respiratory syndrome due to COVID-19. Thus, the aim of this study was to evaluate the possible correlation between vitamin D levels and disease severity in these patients. In consideration of evidence that hypovitaminosis D reduces innate cellular immunity and may stimulate the cytokine storm, which are involved in worsening COVID-19-related ARDS, we also aimed to assess if levels of vitamin D during COVID-19 could further influence the prognosis of those patients.

## Materials and methods

### Data source

This is a retrospective, observational single center study. We obtained the medical records and compiled data from 42 consecutive hospitalized adult inpatients that were admitted to the RICU of the Hospital Policlinic of Bari, Italy, from March 11 through April 30, 2020. Our hospital has been recently transformed in COVID Hospital. Data were collected from doctors working inside the Unit.

The study was approved by the Institutional Review Board of teaching hospital Policlinico of Bari (Ethical Committee No. 6380). The procedures used in this study adhere to the tenets of the Declaration of Helsinki.

COVID-19 diagnosis was based on the WHO interim guidance [9]. A confirmed case of COVID 19 was defined as a positive result on high-throughput sequencing or real-time reverse trascriptase-polymerase-chain reaction (RT-PCR) assay of nasal and pharyngeal swab specimens. Only laboratory confirmed cases, with acute respiratory failure and no need of intubation or invasive ventilation in Intensive Care Unit (ICU) were hospitalized in the RICU and included in this analysis.

Serum 25(OH)D concentration was measured by chemiluminescence immunoassay method, using a Technogenetics kit. Vitamin D insufficiency, moderate deficiency, and severe deficiency were defined as 25(OH)D levels of 20–29, 10–19, and < 10 ng/mL, respectively [12].

### Data collection

Demographic characteristics, medical history, underlined comorbidities, symptoms and signs were collected within the first 12 h following RICU admission. We collected the following data on all patients: laboratory findings, respiratory parameters as fraction of inspired oxygen (FiO2) and arterial partial pressure oxygen PaO_2_/FiO_2_ ratio, respiratory and pharmacological treatment, and outcomes.

### Statistical analysis

Data were expressed as mean and standard deviation (SD) if they were normally distributed while when categorical variables as count (%). We used *T* test to compare data between groups and Pearson correlation to analyze correlation between vitamin D levels, inflammatory indices and respiratory exchange data. We used SPSS software for all analyses. Survival analysis was carried out, comparing the two groups between the first check-up and the final endpoint using the log-rank (Mantel–Cox) test. Significance was established at a *p* value < 0.05.

## Results

The study population included 42 hospitalized patients affected by acute respiratory failure due to COVID-19 and admitted to the RICU.

Baseline demographic and clinical characteristics are described in Table 1. Men were more represented than women (71% vs 29%). Mean age was 65 ± 13 years. Almost all patients were never smokers (52%) or ex-smokers from more than 15 years (43%); two patients only were current smokers (5%). Most of the patients presented at least one comorbidity (86%), with hypertension as the most frequent, followed by cardiovascular diseases, kidney disease, and diabetes. Pulmonary comorbidities were not common: five patients (12%) had chronic obstructive pulmonary disease (COPD) while only two (5%) had asthma. Nine patients (21%) were obese (BMI ≥ 30 kg/m^2^).

**Table 1:** Demographic and clinical characteristics of patients

Patients (*n*)	42
Sex (M/F, *n*, %)	30/12 (71/29)
Age (years, mean, SD)	65 ± 13
BMI (mean, SD)	28,5 ± 5
ARDS (P/F < 300) (*n*, %)	37 (88)
Mild (300 < P/F ≤ 200) (*n*, %)	16 (38)
Moderate (200 < P/F ≤ 100) (*n*, %)	16 (38)
Severe (P/F < 100) (*n*, %)	5 (12)
SOFA score (mean, SD)	3 ± 1.4
Smoking habit (*n*, %)	
Smokers	2 (5)
Ex-smokers	18 (43)
Never smokers	22 (52)
Patients with comorbidity (*n*, %)	36 (86)
Hypertension	26
Cardiovascular disease	16
Chronic kidney disease	16
Diabetes type II	11
Cerebrovascular disease	5
Psycosis, depression, anxiety	10
Malignancy	5
COPD	5
Asthma	2
Vit. D serum level (ng/mL, mean, SD)	20.46 ± 11.6
Non Hypovitaminosis D (vit. D ≥ 30 ng/mL) (*n*, %)	8 (19%)
Hypovitaminosis D (vit. D < 30 ng/mL) (*n*, %)	34 (81%)

*BMI* body mass index, *ARDS* acute respiratory distress syndrome, *SOFA* sequential organ failure assessment, *COPD* chronic obstructive pulmonary disease, *Vit* vitamin

The risk of morbidity and mortality due to sepsis was assessed by Sequential Organ Failure Assessment (SOFA) score (mean 3 ± 1.4) [13]. Most of the patients had severe or very severe hypossiemic respiratory failure: 38% of patients showed a PaO2/FiO2 ratio between 300 and 200, 38% had between 100 and 200, 14% had < 100.

High prevalence of hypovitaminosis D was found in our patients: 81% of patients had vitamin D serum level below 30 ng/mL, with mean serum levels of 20.46 ± 11.6 ng/mL. We also divided patients in four groups by 25(OH)D serum level: vitamin D ≥ 30 ng/mL in patients without hypovitaminosis D (group 1); 30 > vitamin D ≥ 20 ng/mL in patients with insufficiency (group 2); 20 > Vitamin D ≥ 10 ng/mL in patients with moderate deficiency (group 3); vitamin D < 10 ng/mL in patients with severe deficiency (group 4).

The demographic and clinical characteristics of the patients were similar in the four groups (Table 2). Male patients were predominant in all groups. Mean age was higher (74 ± 11 years) in group 4 but this difference did not reach statistical significance. Mean BMI was 31 ± 6.02 kg/m^2^ in patients with moderate vitamin D deficiency, while it was below 30 kg/m^2^ in the remaining three groups.

**Table 2:** Patient’s characteristics based on vitamin D serum level

	Vit. D ≥ 30 ng/mL Group 1	30 > Vit. D ≥ 20 ng/mL (insufficiency) Group 2	20 > Vit. D ≥ 10 ng/mL (moderate deficiency) Group 3	Vit. D < 10 ng/mL (severe deficiency) Group 4
Patients (*n*, %)	8 (19)	11 (26)	13 (31)	10 (24)
Sex (M/F)	3/5	7/4	12/1	8/2
Age (years, mean, SD)	64 ± 18	64 ± 13	60 ± 6.9	74 ± 11
BMI (mean, SD)	27 ± 4	28 ± 4.10	31 ± 6.02	29 ± 4.75
SOFA score (mean, SD)	2 ± 0.74	3 ± 1.81	2 ± 1.51	3 ± 1.06
Smoking habit				
Smokers	0	0	2	0
Ex-smokers	3	4	5	6
Never smokers	5	7	6	4
Patients with comorbidity	6	11	9	10
Patients without comorbidity	2	0	4	0
Comorbidity				
Hypertension	5	8	5	8
Cardiovascular disease	2	6	4	4
Chronic kidney disease	2	6	4	4
Diabetes type II	2	4	3	2
Cerebrovascular disease	0	4	0	1
Psycosis, depression, anxiety	1	4	2	3
Malignancy	1	1	2	1
COPD	1	2	0	2
Asthma	2	0	0	0

*BMI* body mass index, *SOFA* sequential organ failure assessment, *COPD* chronic obstructive pulmonary disease, *Vit* vitamin

The results of laboratory tests in all groups are reported in Table 3. All patients showed high extent of inflammatory serum. We noticed high serum level of CRP, D-dimer and ferritin in all groups. IL-6 serum level tended to be higher (244 ± 468.35 pg/L) in patients with severe vitamin D deficiency but this difference did not reach statistical significance.

**Table 3:** Laboratory test results based on vitamin D serum level

Laboratory test (normal range)	Vit. D ≥ 30 ng/mL (no hypovitaminosis D) Group 1	30 < Vit. D ≥ 20 ng/mL (insufficiency) Group 2	20 < Vit. D ≥ 10 ng/mL (moderate deficiency) Group 3	Vit. D < 10 ng/mL (severe deficiency) Group 4
WBC (4000–10,000 /mm3)	7476 ± 3550	7461 ± 2410	6065 ± 2440	6900 ± 2860
Neutrophils (40–75%/WBC tot)	80 ± 7.88	80 ± 6.67	75 ± 9.99	75 ± 11.86
Lymphocytes (25–55%)	13 ± 6.84	12 ± 5.96	16 ± 7.63	18 ± 10.27
PLT (150–450 103/mm^3^)	278 ± 149.25	177 ± 47.53	238 ± 103.79	207 ± 63.07
CRP (< 2.9)	91 ± 41.74	132 ± 53.14	101 ± 79.93	102 ± 79.98
PCT (0–0.05 ng/mL)	1 ± 0.85	1 ± 1.30	2 ± 4.54	5 ± 11.80
Troponin (> 51.1 pg/mL)	70 ± 112.40	24 ± 20.70	19 ± 17.15	183 ± 381.82
D-dimer (< 500 ug/L)	992 ± 765.27	1726 ± 1621.40	1336 ± 1656.43	2234 ± 1879.81
AST (15–40 U/L)	49 ± 18.31	63 ± 83.38	59 ± 28.73	46 ± 13.20
IL-6 (0–7 pg/L)	83 ± 44.39	83 ± 102.26	54 ± 78.01	244 ± 468.35
Ferritin (8–252 ng/mL)	580 ± 362.36	1154 ± 945.66	573 ± 284,12	943 ± 520.60
LDH (84–246 U/L)	338 ± 50.21	306 ± 70.89	347 ± 99.47	290 ± 75.16
CPK (26–192 U/L)	271 ± 310.51	223 ± 184.89	413 ± 842.67	282 ± 291.06
proBNP (0–166 pg/ml)	763 ± 1118.7	1110 ± 1808.69	159 ± 123.56	2693 ± 4409.08
PaO_2_/FiO_2_ ratio	189 ± 72.89	222 ± 95.42	194 ± 77.77	189 ± 59.12
PaO_2_/FiO_2_ ratio > 300 (number of patients)	1	3	1	0
PaO_2_/FiO_2_ ratio 300–200 (number of patients)	2	3	7	4
PaO_2_/FiO_2_ ratio 200–100 (number of patients)	4	4	3	5
PaO_2_/FiO_2_ ratio < 100 (number of patients)	1	1	2	1

*WBC* white blood cells, *PLT* platelets, *CRP* C-reactive Protein, *PCT* procalcitonin, *AST* aspartate aminotransferase, *IL* interleukin *CPK* creatine phosphokinase, *proBNP* brain natriuretic peptide

Most patients developed ARDS, assessed by PaO_2_/FiO_2_ ratio lower than 300 mmHg. The mean PaO_2_/FiO_2_ ratio was similar between groups.

No statistically significant differences in inflammation indices and respiratory exchange data were found among the four vitamin D groups.

**Fig. 1:**
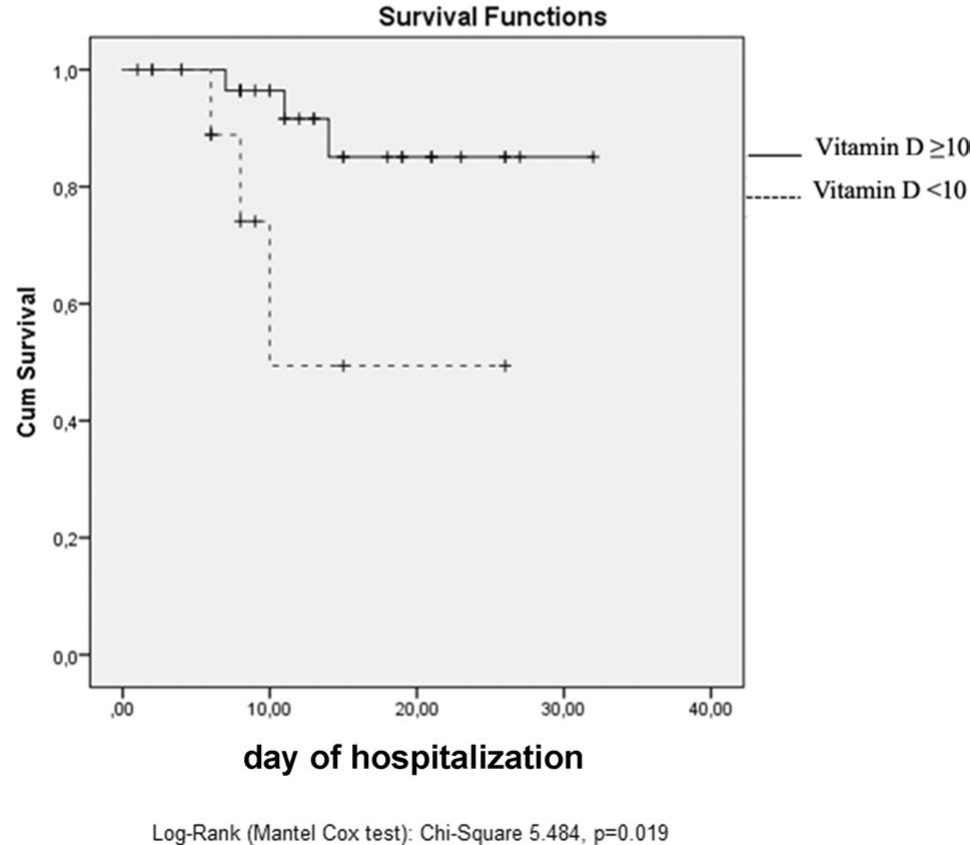
Survival analysis in patients with vitamin D < 10 ng/mL vs patients with vitamin D ≥ 10 ng/mL

Finally, a survival analysis was carried out, comparing patients with vitamin D ≥ 10 ng/mL and those with vitamin D < 10 ng/mL. Patients with severe vitamin D deficiency (*n* = 10) had a median RICU stay of 8 days (IQ25 6, IQ75 11.25), while the remaining 32 patients had a median stay of 12.5 days (IQ 25 8, IQ75 20.5). Indeed, patients with severe vitamin D deficiency tend to have a rapid unfavorable clinical evolution, that results in 20% of cases with death (patients with vitamin D ≥ 10 ng/dL die in 3.1% of cases) and in 20% with transfer to ICU (patients with vitamin D ≥ 10 ng/dL are transferred to ICU in 12.5% of cases). After 10 days, patients with severe vitamin D deficiency had a 50% probability of dying, while those with vitamin D ≥ 10 ng/mL had a 5% mortality risk (*p* = 0.019) (Fig. 1). A COX survival analysis was also performed: in addition to severe vitamin D deficiency, advanced age and higher levels of creatinine, troponin and IL-6 were independent predictors of survival (severe vitamin D deficiency: OR 5681, CI 95% 1114–28,974, *p* 0.037; age: OR 1110, CI 95% 1022–1206, *p* 0.013; troponin: OR 1003, CI 95% 1001–1005, *p* 0.012; IL-6: OR 1004, CI 95% 1001–1006, *p* 0.014; creatinine: OR 1281, CI 95% 1069–1.534, *p* 0.007).

## Discussion

This retrospective observational single center study reports data about the possible association between vitamin D serum level and disease severity and prognosis in patients affected by SARS-CoV-2 with acute respiratory failure.

Since March 2020, the Policlinic of Bari in Italy was transformed in a COVID-19 Hospital. In our RICU, patients referred to Emergency Department with acute respiratory failure due to COVID-19 but without need of intubation and invasive ventilation in ICU were admitted. Our study population was characterized by a mortality risk assessed by SOFA score greater than 36% [13], with high levels of inflammation indices and impaired respiratory exchanges, up to ARDS. In particular, 88% of our patients had mild to severe ARDS, showing a PaO_2_/FiO_2_ ratio between 300 and 200 in 38%, between 100 and 200 in 38%, and < 100 in 14% of patients.

In these patients, we found high prevalence of hypovitaminosis D (81%). Twenty-four percent of patients had severe deficiency, showing vitamin D serum level below than 10 ng/mL. In the position statement of the European Calcified Tissue Society, it is reported that vitamin D deficiency is common in Europe, especially in Western, Southern and Eastern Europe (30–60%) and in Middle East countries (> 80%), and that severe deficiency is found in > 10% of Europeans. In addition to sun exposure and dietary intake, age over 70 years and institutionalization are presented as the main risk factors for impaired vitamin D status [14]. Our study was conducted during the quarantine period, in the winter months, in patients with an average age over 65 years, some of whom were institutionalized; all these factors could explain our results.

Based on vitamin D serum levels we divided our cohort into four groups: 19% of patients had no hypovitaminosis D, 26% had insufficiency, 31% had moderate deficiency, and 24% had severe deficiency. No statistically significant differences were found between the groups regarding inflammation indexes and respiratory exchanges data. IL-6 levels are higher in patients with severe vitamin D deficiency but this data do not reach statistical significance. This might be related to the sample size, therefore analysis on a larger number of patients would be needed. By contrast, a survival analysis showed that, after 10 days of hospitalization, patients with severe vitamin D deficiency had a significantly higher mortality risk than the others. This finding may suggest a possible role for the vitamin D supplementation in the supportive treatment of COVID-19 patients.

These data are in line with results of a study conducted by Ilie et al. They focused on mean vitamin D levels in European countries (severely low in aging population especially in Italy, Spain and Switzerland) and observed a negative correlation between vitamin D levels and COVID-19 cases and mortality in the population of those countries [15]. Accordingly, in a recent study, Grant et al. reported that vitamin D could reduce the risk of influenza and COVID-19. The outbreak occurred in winter, a time when vitamin D concentrations are lowest; on the other hand, the number of cases in the Southern Hemisphere near the end of summer are low. Vitamin D deficiency has been found to contribute to ADRS; case-fatality rates increase with age and with chronic disease comorbidity, both of which are associated with lower 25(OH)D concentration.

Altogether, these considerations support the recommendation that people at risk of influenza and/or COVID-19 consider vitamin D supplementation to raise their 25(OH)D concentrations above 40–60 ng/mL, and that treatment of patients infected with influenza and/or COVID-19 includes higher vitamin D doses. However, randomized controlled trials and large population studies should be conducted to evaluate these recommendations specifically in COVID-19 patients [8].

Furthermore, in a recent review, Panfili and colleagues report some evidence of a role of vitamin D in preventing lung fibrosis, described as a common complication of ARDS and that could be a long-term issue in these patients. This antifibrotic role could be an additional element supporting the use of vitamin D supplementation before and after COVID 19 infection. However, this hypothesis will be supported by studies with longer follow-up on survivors [16].

The association between hypovitaminosis D, airways inflammation and increased risk of respiratory infections started before the COVID-19 era when a large body of studies evaluated the efficacy of vitamin D supplementation as adjunctive treatment in patients with respiratory diseases. Lehouck et al. explored the role of supplementation with high doses of vitamin D in reducing the incidence of COPD exacerbations. In that study, patients with moderate to very severe COPD and a history of recent exacerbations were randomized to receive 100,000 IU of vitamin D supplementation or placebo every 4 weeks for 1 year. No differences in terms of median time to first exacerbation, exacerbation rate, FEV1, hospitalization, quality of life and death were found between the vitamin D and placebo groups. However, a post hoc analysis conducted on patients with severe vitamin D deficiency (< 10 ng/mL) showed a significant reduction in exacerbation rate in vitamin D group [17].

Moreover, a meta-analysis conducted by Martineau et al. on 25 RCTs highlighted that vitamin D supplementation reduced the risk of acute respiratory infections, and that the protective vitamin D effects were more evident in those individuals with 25(OH)D baseline concentrations below 25 nmol/L at baseline [18]. In our study, a higher mortality risk was related to lower 25(OH)D levels.

In addition, along with age and levels of troponin, creatinine and IL-6, severe vitamin D deficiency emerged as an independent survival factor in COVID-19 patients. This suggest that vitamin D supplementation may not protect against COVID-19 infections, but, in case of infection, may reduce the severity of the disease and consequently the risk of death.

In our RICU, no correlation between inflammations indices, respiratory exchanges data and vitamin D serum level was found. This might be related with the severe clinical conditions of these patients, characterized by moderate to severe hypoxemic respiratory failure, requiring treatment with non-invasive mechanical ventilation or high-flow oxygen. Moreover, in our population the large majority was already characterized by hypovitaminosis D. Nevertheless, the lack of correlation between inflammatory indices and vitamin D levels may be related to sample size but it may also suggest that severe vitamin D deficiency may influence outcomes independently of its immunomodulatory properties.

This study presented some limitations. Firstly, the sample size that is modest. As previously mentioned, this may have limited the results of statistical analyzes, especially those conducted within the cited four subgroups. The classification in four groups was used according to clinical practice, in which different levels of hypovitaminosis D provide for different therapeutic dosages. Secondly, the relatively short follow-up of patients enrolled, related to hospitalization times in a RICU. Indeed, patients admitted to our RICU for acute respiratory failure due to COVID-19, were transferred to a lower-intensity care wards, in case of stabilization of clinical conditions, or to ICU, in case of worsening respiratory failure. This aspect, associated with the severity of the clinical conditions of some patients, reduced hospitalization times and consequently patients follow-up.

In conclusion, the results of our study show a high prevalence of hypovitaminosis D in COVID-19 patients treated in a RICU. Higher risk of mortality was found in patients with severe vitamin D deficiency. Further studies need to be conducted on a larger population, to demonstrate whether adjunctive treatment with vitamin D might be effective in improving disease outcomes and in reducing mortality risk.

## Data Availability

The datasets generated during and/or analysed during the current study are available from the corresponding author on reasonable request.
